# Future approaches to clearing the latent human immunodeficiency virus reservoir: Beyond latency reversal

**DOI:** 10.4102/sajhivmed.v21i1.1089

**Published:** 2020-08-12

**Authors:** Alexander M.L. Hayes

**Affiliations:** 1 Medical Sciences Division, Faculty of Clinical Medicine, University of Oxford, Oxford, United Kingdom

**Keywords:** HIV, latency reversal, viral reservoir, pro-apoptotic drugs, cART

## Abstract

**Background:**

While combined antiretroviral therapy (cART) allows near-normal life expectancy for people living with human immunodeficiency virus (HIV), it is unable to cure the infection and so life long treatment is required.

**Objectives:**

The main barrier to curing HIV is the latent reservoir of cells, which is stable and resistant to cART.

**Method:**

Current approaches under investigation for clearing this reservoir propose a ‘Shock and Kill’ mechanism, in which active replication is induced in latent cells by latency reversal agents, theoretically allowing killing of the newly active cells.

**Results:**

However, previous studies have failed to achieve depletion of the *T* central memory cell reservoir, are unable to target other latent reservoirs and may be causing neurological damage to participants.

**Conclusion:**

Future approaches to clearing the latent reservoir may bypass latency reversal through the use of drugs that selectively induce apoptosis in infected cells. Several classes of these pro-apoptotic drugs have shown promise in *in vitro* and *ex vivo* studies, and may represent the basis of a future functional cure for HIV.

## Introduction

The global HIV/AIDS pandemic has caused over 25 million deaths since its origins in the 20th century and remains without a cure. Combined antiretroviral therapy (cART) is the current treatment of choice for human immunodeficiency virus (HIV) infection^1^ and suppresses viral load below detectable levels with correct administration.^1,2^ However, lifelong treatment is required, at considerable expense to healthcare systems,^1^ as the cessation of therapy causes a rapid rebound of viraemia within days^3^ and eventual progression of the disease to AIDS. Furthermore, escape mutations of the virus are common and reduce efficacy,^4,5^ necessitating expensive combination therapy with multiple drugs. Viral rebound occurs due to the presence of latent viral reservoirs, rather than continuing low-level replication during cART, as shown by clonal evolutionary studies.^6^ On therapy cessation these latently infected cells clonally expand, seeding a population of infected cells containing intact provirus.^7^ Whilst cART has been successful in extending life expectancy of people living with HIV in high-income countries, this remains impaired compared to healthy adults.^8^ As cART is limited by mutations, is an expensive lifelong treatment and may not be available to those in countries with poorly developed healthcare systems, development of a cure for HIV is desirable. Cure research would be of considerable impact in South Africa, which has the highest number of people living with HIV in the world, at 7.7 m as of 2018, a prevalence of 20.4% in adults (UNAIDS 2019)^9^. In that year 77 000 deaths were recorded due to AIDS-related illnesses (UNAIDS 2019), although this number is coming down and life expectancy is rising as the cART programme continues to grow.

Reservoirs of latently infected cells are the main barrier to cART curing HIV. The first latently infected cells to be identified were CD4^+^ central memory T cells (T_CM_) containing replication-competent, integrated provirus.^10^ These were found to be present in patients on cART, with no change in reservoir size with continuous therapy^11^ as the quiescent cells do not contain actively replicating virus, and so are unaffected by cART. The T_CM-_ reservoir is present even if cART is initiated within the first week post exposure,^12^ so is established early in infection. It is also extremely stable, as the latent cells have a half-life of around 44 months,^13^ so is unlikely to be cleared in a patient’s lifetime. The T_CM-_ reservoir contains the majority of latent cells, but other reservoirs are also present and form barriers to cure research. Several myeloid cell lines have been shown to harbour latent HIV in chronic infection and contribute to viral rebound on cART cessation.^14,15^ There is evidence for the presence of latent reservoirs in tissue macrophages^16^ and microglial cells and astrocytes in the central nervous system (CNS).^17,18^ There is no *in vivo* evidence of latently infected tissue dendritic cells but infection has been shown *in vitro*,^19^ and it has been proposed that this may be transferred to CD4^+^ cells via a virological synapse.^20^ Follicular dendritic cells have been shown to maintain a stable pool of virus on their surface without being infected, providing a latent reservoir within secondary lymphoid tissue.^21^ Further reservoirs have been suggested, such as within fibrocytes,^20^ but it is now apparent that the T_CM_ reservoir is not the only source of latently infected cells. Potential cures must, therefore, target all identified reservoirs to be successful.

Several approaches to clearing the latent reservoirs have been investigated, with the ‘Shock and Kill’ combination receiving particular interest. However, recent findings seem to suggest this is unlikely to ever provide a cure for HIV^22,23,24,25,26,27,28,29,30^ and may have unresearched deleterious side effects.^31,32,33^ An emerging class of drugs targeting the apoptosis pathways in latently infected cells may allow reservoir depletion without latency reversal, and therefore merit further research as a potential cure for HIV.

## Shock and Kill

**FIGURE 1:**
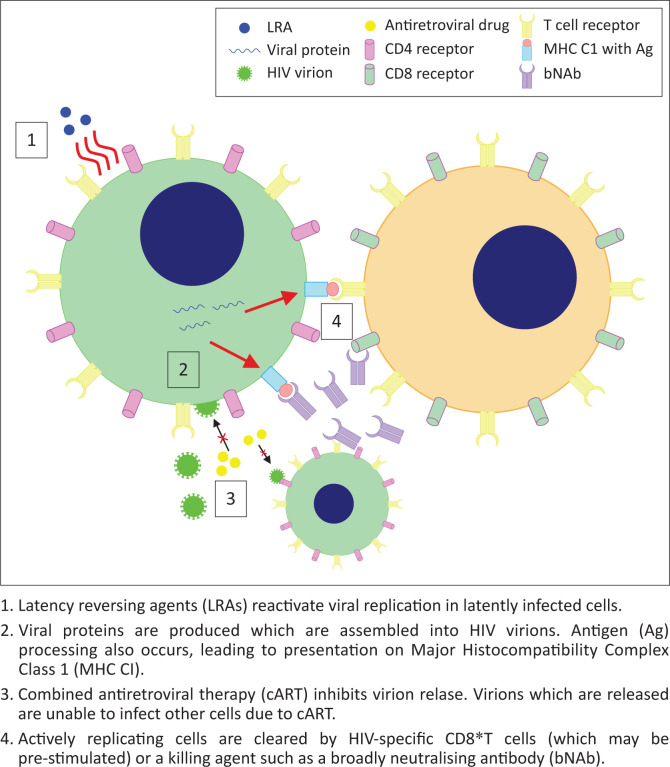
Proposed mechanism of ‘Shock and Kill’.

A potential approach to clearing HIV latent reservoirs is ‘Shock and Kill’, in which the reservoir would be ‘shocked’ with latency-reversing agents (LRAs) to induce replication of the provirus. ‘Killing’ of the activated cells would then be achieved through viral cytopathic effects, CD8^+^ T cells, cART or other agent, resulting in clearance of the latent cells and curing the infection. This mechanism is outlined in Figure 1. Several LRAs have been developed, many using Yang’s *in vitro* model of latently infected CD4^+^ T_CM_^34^ to identify compounds that selectively induce viral replication in latent cells. These include disulfiram^22^ and histone deacetylase inhibitors (HDACIs) such as vorinostat and panobionstat. Early *in vivo* studies were promising as latency reversal was demonstrated with an increase in viral gene expression in the resting cells observed with disulfiram,^23,24^ vorinostat^25^ and panbionstat^26^ administration. However, the key caveat is that none of these studies decreased reservoir size *in vivo* as the number of latent cells did not decrease following latency reversal. Whilst replication was induced in a proportion of the latent cells, this did not lead to immune or viral-mediated killing of reactivated cells.

Several reasons have been proposed for the failure of LRAs alone to reduce reservoir size. The initial principle of ‘Shock and Kill’ was that viral cytopathic effects and lysis, due to HIV replication, aided by cytotoxic T lymphocyte (CTL)-mediated killing of actively infected cells, would be sufficient to kill reactivated, formerly latent, cells. Shan et al.^27^ showed that the viral cytopathic effect alone is insufficient to kill cells, and the vigorous CTL response is lost in chronic HIV infection. However, CTL stimulation increased latent cell clearance, leading to the suggestion that some form of immune stimulation may be required alongside latency reversal to achieve a reduction in reservoir size. The CTL response to reactivated cells may be insufficient to reduce reservoir size on latency reversal, due to 98% of latent T cells carrying escape mutations to CTL killing^28^ and T cell exhaustion in chronic infection^29^ reducing the efficacy of the CTL response. Furthermore, LRAs may not affect non-T_CM-_ reservoirs^30^ as macrophages are resistant to the cytopathic effects of HIV, and dendritic cells do not integrate viral DNA, meaning that LRAs would be unable to cause viral replication and subsequent targeting by the immune system. It has also been proposed that continued cART may not completely inhibit infection of further cells by virions produced from reactivated, formerly latent cells.^30^ Thus, LRA administration may lead to infection of previously uninfected cells. It is therefore clear that LRA administration alone does not reduce reservoir size, due to a lack of viral cytopathic effects and lysis, insufficient CTL response and the potential failure of cART to suppress replication. As such, stages 3 and 4 from Figure 1 are unlikely to be successful and an additional killing agent is required to achieve the second goal of ‘Shock and Kill’. This may involve immune stimulation, with natural killer (NK) cell stimulation showing some promise *ex vivo*^35^ or other agents, such as broadly neutralising antibodies (bNAbs), which appear to mediate antibody-dependent cellular cytotoxicity of activated latently infected cells in mice^36^ but human studies are required to assess their efficacy. Killing agents that selectively induce apoptosis of HIV-infected cells have also shown promise *in vitro* (see Pro-apoptotic drugs [PADs]).

## Limitations of latency-reversing agents

Whilst it has been repeatedly shown that LRAs alone are unable to reduce the size of the latent HIV reservoir, administration of a killing agent alongside an LRA has been proposed as a method of clearing the reactivated cells.^37^ However, it seems likely that even with killing agents LRAs cannot clear enough latent cells to achieve a functional cure and may cause complications due to non-T cell HIV reservoirs. Future studies in humans to test the efficacy of killing agents administrated alongside LRAs could be carried out by administering a previously tested LRA, such as disulfiram, alongside a killing agent such as a bNAb to participants living with HIV and measuring the change in reservoir size using a technique such as TILDA.^38^ However, the combination of an LRA with, for example, a bNAb, is unlikely to clear the myeloid reservoir as dendritic cells are likely to be unaffected by LRAs.^30^ Therefore, even if functional elimination of the T_CM_ reservoir is achieved, viral rebound on cART cessation may still occur due to the presence of the myeloid reservoir, which would likely not be cleared by current ‘Shock and Kill’ approaches under investigation.

**FIGURE 2:**
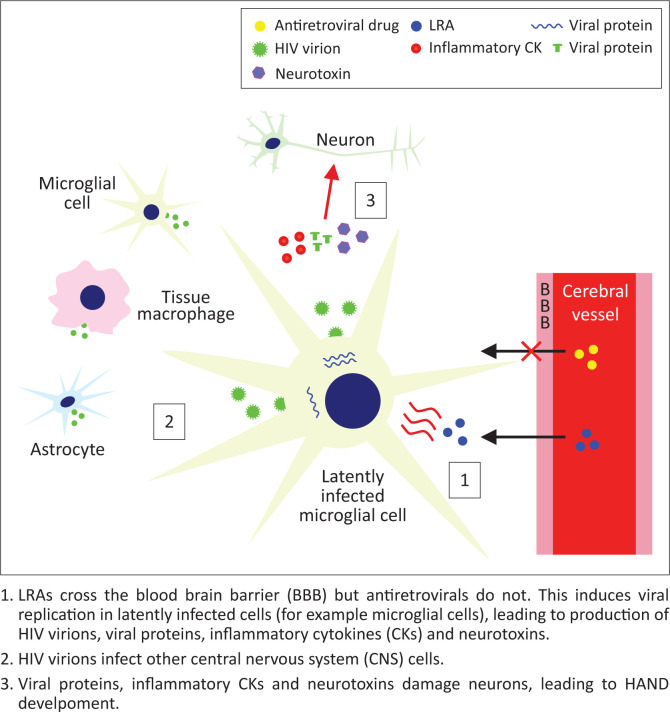
Proposed mechanism for human immunodeficiency virus associated neurological disorder development on latency reversing agent administration.

Human immunodeficiency virus-associated neurological disorders (HANDs) are well-observed in patients on cART and may lead to widespread neurological impairment,^39^ suggesting significant damage to the CNS even when viraemia elsewhere is suppressed below detectable levels. The cause of this appears to be relatively poor blood-brain barrier (BBB) penetrance by antiretrovirals,^31^ allowing continued HIV replication alongside the presence of latently infected microglial cells, perivascular macrophages and astrocytes.^20^ Recent research suggests that impaired neurogenesis may underpin HAND persistence even with cART.^32^ A study on the CNS penetrance of LRAs^33^ showed that disulfiram and vorinostat are able to cross the BBB relatively easily. Whilst this could enable targeting of the CNS latent reservoir, it may mean that current *in vivo* studies of LRAs are damaging to the patients involved, as there is no reduction in latent reservoir size, whilst LRAs are able to penetrate the CNS and increase viral replication with no inhibition from antiretroviral drugs. This may potentially lead to an increase in viral protein, inflammatory cytokine (CK) and neurotoxin release from infected macrophages and microglia, which could increase the probability of HAND development (personal communication, Grant Campbell) as shown in Figure 2. Therefore, in addition to their inability to affect all HIV reservoirs, even with the accompanying administration of killing agents, LRAs may cause increased neurological impairment of patients. Furthermore, if the killing agent used in a hypothetical cure is unable to penetrate the BBB, there would be no reduction in the size of the CNS reservoir. This makes the development of a cure strategy distinct from ‘Shock and Kill’, involving the direct killing of latent cells without the need for latency reversal, desirable. The PAD class mentioned earlier has shown promise in achieving this.

## Interactions of human immunodeficiency virus with apoptosis

The human immunodeficiency virus has complex interactions with apoptosis pathways in infected cells.^40^ In latently infected cells the increase in longevity observed is partially the result of avoidance of apoptosis.^37^ A number of specific effects on apoptosis pathways have been suggested as the cause of this. The intrinsic apoptosis pathway is a response to cellular damage and involves pro-apoptotic factors causing mitochondrial outer membrane permeabilisation (MOMP), triggering the release of soluble proteins from the mitochondrial intermembrane space. These include the cytochrome c and second mitochondria-derived activator of caspase (SMAC, also known as DIABLO^41^). Cytochrome c binds cytosolic proteins to form the apoptosome, which activates the ‘initiator’ enzyme caspase-9. This, in turn, cleaves the ‘executioner’ enzymes caspase-3 and caspase-7 which trigger the cleavage cascade of other caspase enzymes resulting in cell death. The second mitochondria-derived activator of caspase activates this process through disinhibition of caspases 3, 7 and 9 via binding and inactivating the X-linked inhibitor of apoptosis (XIAP). The X-linked inhibitor of apoptosis binds these caspases in the absence of SMAC, inhibiting their activity and directing their degradation by the proteasome. The second mitochondria-derived activator of caspase antagonises this activity, allowing the progression of the apoptotic process.^41^ Several other IAPs are also inhibited by SMAC, including the baculoviral IAP repeat-containing protein 2 (BIRC, also known as cellular IAP1).^41^

Viral proteins, in particular Tat and Nef, cause inhibition of this intrinsic apoptosis pathway in latently infected cells. Tat upregulates host production of anti-apoptotic proteins including XIAP and Bcl2.^42^ The X-linked inhibitor of apoptosis inhibits caspase activity, as detailed earlier, whilst Bcl2 inhibits MOMP,^43^ thus preventing the release of cytochrome c and SMAC. Tat also inhibits the pro-apoptotic proteins p53 and the Bcl2-associated antagonist of cell death (BAD).^40^ Nef causes p21-activated kinase activation, leading to phosphorylation of BAD, thus inhibiting its pro-apoptotic activity (binding to and inhibition of Bcl2),^44^ and also inhibits caspase-8 and caspase-10.^40^ Conversely, in the CNS, it appears that Tat and Nef may induce autophagy and apoptosis in HAND pathogenesis.^45,46^ Expression and activation of anti-apoptotic proteins are therefore increased in latently infected cells, making this an attractive therapeutic target.

## Pro-apoptotic drugs

Several classes of pro-apoptotic compounds have been developed recently for cancer treatment,^47,48^ as tumour cells often over-express or activate IAPs allowing the evasion of apoptosis, a key hallmark of cancer. Some of these have been shown to induce apoptosis of latent HIV infected cells *in vitro*, leading to speculation that PADs may contribute to a future cure.

Second mitochondria-derived activator of caspase mimetics (SMs) are a class of drugs which bind IAPs in a similar fashion to the endogenous molecule.^48^ They cause caspase activation via IAP binding^49^ and appear to reduce IAP activity through both degradation and inhibition of the proteins,^50^ and therefore induce apoptosis. Second mitochondrias have recently been developed for cancer treatment, inducing apoptosis^48^ of tumour cells which over-express or activate IAPs. The SMs birinapant, embelin and GDC-0152 were investigated for their efficacy to induce apoptosis in latently infected T_CM_ by Campbell et al.^51^ All were found to cause rapid degradation of XIAP and BIRC2, which were upregulated in the infected cells, and importantly showed selective killing of HIV-infected T_CM_ compared to uninfected T_CM_, with the doses required for 90% clearance of HIV-infected cells causing a small increase of T_CM_ cell death, of 3.5% – 4.6% above basal levels. This occurred in the absence of increased virus production, despite findings in models of latency by Pache et al.^52^ suggesting that SMs may act as LRAs due to the reduction in BIRC2 leading to increased NFκβ signalling, resulting in an increased viral transcription. However, in latent HIV-T_CM_ from patients undergoing cART, the Pache study showed that SMs only caused latency reversal when used in combination with an LRA such as vorinostat. Therefore, it appears that the major effect of SMs on latently infected T_CM- -_is to induce apoptosis rather than reactivate transcription.

Furthermore, Campbell et al. demonstrated the mechanism of apoptosis on SM administration is dependent on the induction (but not completion) of autophagy. Wortmannin (an inhibitor of early stages of autophagy) led to a reduction in apoptosis despite the degradation of IAPs by SMs. Further findings suggest that, following IAP degradation, autophagy proteins form a scaffold for the assembly of a ripoptosome-like death-inducing signaling complex (DISC), which can then initiate apoptosis via caspase 8 induction. This is an additional mechanism to the simple removal of IAP inhibition due to their degradation and may explain the selectivity of SMs for inducing death of HIV-T_CM_. Inhibitors of later stages of autophagy did not reduce apoptosis, suggesting that the process of autophagy itself is not required for this mechanism of cell death. Induction of autophagy is sufficient for apoptosis, as autophagy proteins allow DISC formation. IAPs are degraded by SMs in uninfected T_CM_ but this does not lead to the same degree of cell death due to reduced autophagy induction and therefore decreased probability of DISC formation. HIV-T_CM_ are more prone to autophagy induction than uninfected cells as a result of the effects of viral proteins. Gag and Nef interact with autophagy factors to increase autophagy induction, which in turn augments HIV yields in cells containing the actively replicating virus.^53^ Nef and Vif inhibit later stages of autophagy to prevent HIV degradation.^53,54^ Therefore, HIV-T_CM_ are more prone to autophagy induction than uninfected cells, providing a potential explanation for the selective apoptosis observed in latently infected cells on SM administration. Over-expression or activation of IAPs in infected cells may also contribute to the selectivity of SMs.

These findings present *ex vivo* evidence of the selective killing of latently infected T_CM_ by SMs, along with a convincing mechanism of how uninfected cells are spared. Clinical trials in humans may now be justified, but should be performed with caution as the side effects of these drugs in people living with HIV are unknown. A number of SMs have been used in phase I trials for various cancers, with a generally tolerable safety profile, although the occasional occurrence of cytokine release syndrome in patients is of some concern.^55^ *In vitro* and *ex vivo* findings suggest SMs may be able to deplete the T_CM_ reservoir *in vivo*, and, whilst if 90% of latently infected cells are killed (as in the Campbell study) the patient will not be cured of HIV, this eclipses reservoir clearance demonstrated by current ‘Shock and Kill’ approaches. However, it is unclear if these drugs would induce apoptosis in non-T cell reservoirs to the same degree, which may prevent the development of a functional cure. Future *in vitro* and *ex vivo* studies of PADs using latently infected myeloid cells from people living with HIV would be advisable to further test their suitability.

*In vitro* and *ex vivo* studies of other PADs have also shown promise. Bcl-2 antagonists such as venetoclax caused disinhibition of MOMP and led to the depletion of latent HIV-T_CM_ from patients on cART in an *ex vivo* study.^56^ P13K/Akt inhibitors increase the expression of pro-apoptotic genes downregulated by HIV and have shown promise in predisposing latently infected macrophages and microglia to cell death.^57,58^ RIG-1 inducers activate an innate immune response to viral RNA, triggering apoptosis. An *ex vivo* study demonstrated selective depletion of latent HIV-T_CM_ using the RIG-1 inducer acitretin.^59^ These classes of PADs also merit further investigation and could be used in combination with SMs in a future approach to clearing the latent reservoir to induce apoptosis in a greater portion of latently infected cells.

## Conclusions

Recent ‘Shock and Kill’ research has failed to demonstrate significant depletion of the viral reservoir, and it remains unclear if the use of LRAs may cause HANDs. PADs may represent a promising future approach to curing HIV given their *in vitro* and *ex vivo* success in selectively causing apoptosis in latently infected cells, with SMs appearing effective against the T_CM_ reservoir and P13K/Akt inhibitors against myeloid reservoirs. Several of these drugs have been used in clinical trials for other conditions, and further investigation into their potential for people living with HIV is merited.

## References

[1] Volberding PA, Deeks SG Antiretroviral therapy and management of HIV infection. Lancet. 2010;376(9734):49–62. 10.1016/s0140-6736(10)60676-9 20609987

[2] Palmer S, Maldarelli F, Wiegand A, et al Low-level viremia persists for at least 7 years in patients onsuppressive antiretroviral therapy. Proc Natl Acad Sci U S A. 2008;105(10):3879–3884. 10.1073/pnas.0800050105 18332425 PMC2268833

[3] Fischer M, Hafner R, Schneider C, et al HIV RNA in plasma rebounds within days during structuredtreatment interruptions. AIDS. 2003;17(2):195–199. 10.1097/01.aids.0000042945.95433.4b 12545079

[4] Poggensee G, Kücherer C, Werning J, et al Impact of transmission of drug-resistant HIV on the course of infection and the treatment success. Data from the German HIV-1 seroconverter study. HIV Med. 2007;8(8):511–519. 10.1111/j.1468-1293.2007.00504.x 17944684

[5] Wittkop L, Günthard H, de Wolf F, et al Effect of transmitted drug resistance on virological and immunological response to initial combination antiretroviral therapy for HIV (EuroCoord-CHAIN joint project): A European multicohort study. Lancet Infect Dis. 2011;11(5):363–371. 10.1016/S1473-3099(11)70032-9 21354861

[6] Joos B, Fischer M, Kuster H, et al HIV rebounds from latently infected cells, rather than from continuing low-level replication. Proc Natl Acad Sci U S A. 2008;105(43):16725–16730. 10.1073/pnas.0804192105 18936487 PMC2575487

[7] Simonetti FR, Sobolewski M, Fyne E, et al Clonally expanded CD4+ T cells can produce infectious HIV-1 invivo. Proc Natl Acad Sci U S A. 2016;113(7):1883–1888. 10.1073/pnas.1522675113 26858442 PMC4763755

[8] Collaboration A TC Life expectancy of individuals on combination antiretroviral therapy in high-income countries: A collaborative analysis of 14 cohort studies. Lancet. 2008;372(9635):293–299. 10.1016/S0140-6736(08)61113-7 18657708 PMC3130543

[9] UNAIDS website: https://www.unaids.org/en/regionscountries/countries/southafrica

[10] Chun TW, Stuyver L, Mizell S, et al Presence of an inducible HIV-1 latent reservoir during highly active antiretroviral therapy. Proc Natl Acad Sci U S A. 1997;94(24):13193–13197. 10.1073/pnas.94.24.13193 9371822 PMC24285

[11] Finzi D, Hermankova M, Pierson T, et al Identification of a reservoir for HIV-1 in patients on highly active antiretroviral therapy. Science. 1997;278(5341):1295–1300. 10.1126/science.278.5341.1295 9360927

[12] Chun TW, Engel D, Berrey M, et al Early establishment of a pool of latently infected, resting CD4(+) T cells during primary HIV-1 infection. Proc Natl Acad Sci U S A. 1998;95(15):8869–8873. 10.1073/pnas.95.15.8869 9671771 PMC21169

[13] Siliciano JD, Kajdas J, Finzi D, et al Long-term follow-up studies confirm the stability of the latent reservoir for HIV-1 in resting CD4+ T cells. Nat Med. 2003;9(1):727–728. 10.1038/nm880 12754504

[14] Abbas W, Tariq M, Iqbal M, Kumar A, Herbein G Eradication of HIV-1 from the macrophage reservoir: An uncertain goal? Viruses. 2015;7(4):1578–1598. 10.3390/v7041578 25835530 PMC4411666

[15] Gama L, Abreu C, Shirk E, et al SIV Latency in macrophages in the CNS. Curr Top Microbiol Immunol. 2018;417:111–130. 10.1007/82_2018_89 29770863 PMC6468993

[16] Honeycutt JB, Thayer W, Baker C, et al HIV persistence in tissue macrophages of humanized myeloidonly mice during antiretroviral therapy. Nat Med. 2017;23:638–643. 10.1038/nm.4319 28414330 PMC5419854

[17] Churchill MJ, Gorry P, Cowley D, et al Use of laser capture microdissection to detect integrated HIV-1 DNA in macrophages and astrocytes from autopsy brain tissues. J Neurovirol. 2006;12:146–152. 10.1080/13550280600748946 16798676

[18] Kramer-Hämmerle S, Rothenaigner I, Wolff H, Bell JE, Brack-Werner R Cells of the central nervous system as targets and reservoirs of the human immunodeficiency virus. Virus Res. 2005;111(2):194–213. 10.1016/j.virusres.2005.04.009 15885841

[19] Kawamura T, Gulden F, Sugaya M, et al R5 HIV productively infects Langerhans cells, and infection levels are regulated by compound CCR5 polymorphisms. Proc Natl Acad Sci U S A. 2003;100(14):8401–8406. 10.1073/pnas.1432450100 12815099 PMC166241

[20] Kandathil AJ, Sugawara S, Balagopal A Are T cells the only HIV-1 reservoir? Retrovirology. 2016;13:86 10.1186/s12977-016-0323-4 27998285 PMC5175311

[21] Spiegel H, Herbst H, Niedobitek G, Foss HD, Stein H Follicular dendritic cells are a major reservoir for human immunodeficiency virus type 1 in lymphoid tissues facilitating infection of CD4+ T-helper cells. Am J Pathol. 1992;140:15–22. 1530997 PMC1886262

[22] Xing S, Bullen C, Shroff N, et al Disulfiram reactivates latent HIV-1 in a Bcl-2-transduced primary CD4+ T cell model without inducing global T cell activation. J Virol. 2011;85(12):6060–6064. 10.1128/JVI.02033-10 21471244 PMC3126325

[23] Spivak AM, Andrade A, Eisele E, et al A pilot study assessing the safety and latency-reversing activity of disulfiram in HIV-1-infected adults on antiretroviral therapy. Clin Infect Dis. 2014;58(6):883–890. 10.1093/cid/cit813 24336828 PMC3935499

[24] Elliott JH, McMahon J, Chang C, et al Short-term administration of disulfiram for reversal of latent HIV infection: A phase 2 dose-escalation study. Lancet HIV. 2015;2(12):e520–529. 10.1016/S2352-3018(15)00226-X PMC5108570 26614966

[25] Archin NM, Liberty A, Kashuba A et al Administration of vorinostat disrupts HIV-1 latency in patients on antiretroviral therapy. Nature. 2012;487:482–485. 10.1038/nature11286 22837004 PMC3704185

[26] Rasmussen TA, Tolstrup M, Brinkmann C, et al Panobinostat, a histone deacetylase inhibitor, for latent-virus reactivation in HIV-infected patients on suppressive antiretroviral therapy: A phase 1/2, single group, clinical trial. Lancet HIV. 2014;1(1):e13–e21. 10.1016/S2352-3018(14)70014-1 26423811

[27] Shan L, Deng K, Shroff N, et al Stimulation of HIV-1-specific cytolytic T lymphocytes facilitates elimination of latent viral reservoir after virus reactivation. Immunity. 2012;36(3):491–501. 10.1016/j.immuni.2012.01.014 22406268 PMC3501645

[28] Deng K, Pertea M, Rongvaux A, et al Broad CTL response is required to clear latent HIV-1 due to dominance of escape mutations. Nature. 2015;517:381–385. 10.1038/nature14053 25561180 PMC4406054

[29] Chew GM, Fujita T, Webb G, et al TIGIT marks exhausted T cells, correlates with disease progression, and serves as a target for immune restoration in HIV and SIV infection. PLoS Pathog. 2016;12:e1005349 10.1371/journal.ppat.1005349 26741490 PMC4704737

[30] Thorlund K, Horwitz MS, Fife BT, Lester R, Cameron DW Landscape review of current HIV ‘kick and kill’ cure research – Some kicking, not enough killing. BMC Infect Dis. 2017;17:595 10.1186/s12879-017-2683-3 28851294 PMC5576299

[31] Letendre S, Marquie-Beck J, Capparelli E, et al Validation of the CNS penetration-effectiveness rank for quantifying antiretroviral penetration into the central nervous system. Arch Neurol. 2008;65(1):65–70. 10.1001/archneurol.2007.31 18195140 PMC2763187

[32] Putatunda L, Ho WZ, Hu W HIV-1 and compromised adult neurogenesis: Emerging evidence for a new paradigm of HAND persistence. AIDS Rev. 2019;21:11–22. 10.24875/AIDSRev.19000003 30899112 PMC6911301

[33] Churchill MJ, Cowley DJ, Wesselingh SL, Gorry PR, Gray LR HIV-1 transcriptional regulation in the central nervous system and implications for HIV cure research. J Neurovirol. 2015;21:290–300. 10.1007/s13365-014-0271-5 25060300 PMC4305497

[34] Yang HC, Xing S, Shan L, et al Small-molecule screening using a human primary cell model of HIV latency identifies compounds that reverse latency without cellular activation. J Clin Invest. 2009;119:3473–3486. 10.1172/JCI39199 19805909 PMC2769176

[35] Garrido C, Abad-Fernandez M, Tuyishime M, et al Interleukin-15-stimulated natural killer cells clear HIV-1-infected cells following latency reversal ex vivo. J Virol. 2018;92(12):e00235–18. 10.1128/JVI.00235-18 29593039 PMC5974478

[36] Halper-Stromberg A, Lu C, Klein F, et al Broadly neutralizing antibodies and viral inducers decrease rebound from HIV-1 latent reservoirs in humanized mice. Cell. 2014;158(5):989–999. 10.1016/j.cell.2014.07.043 25131989 PMC4163911

[37] Kim Y, Anderson JL, Lewin SR Getting the ‘Kill’ into ‘Shock and Kill’: Strategies to eliminate latent HIV. Cell Host Microbe. 2018;23(1):14–26. 10.1016/j.chom.2017.12.004 29324227 PMC5990418

[38] Procopio FA, Fromentin R, Kulpa D, et al A novel assay to measure the magnitude of the inducible viral reservoir in HIV-infected individuals. EBioMedicine. 2015;2(8):874–883. 10.1016/j.ebiom.2015.06.019 26425694 PMC4563128

[39] Heaton RK, Clifford DB, Franklin DR, et al HIV-associated neurocognitive disorders persist in the era of potent antiretroviral therapy: CHARTER study. Neurology. 2010;75(23):2087–2096. 10.1212/WNL.0b013e318200d727 21135382 PMC2995535

[40] Timilsina U, Gaur R Modulation of apoptosis and viral latency – An axis to be well understood for successful cure of human immunodeficiency virus. J Gen Virol. 2016;97(4):813–824. 10.1099/jgv.0.000402 26764023

[41] Green DR, Llambi F Cell death signaling. Cold Spring Harb Perspect Biol. 2015;7 10.1101/cshperspect.a006080 PMC4665079 26626938

[42] López-Huertas MR, Mateos E, Sánchez del Cojo M et al The presence of HIV-1 Tat protein second exon delays fas protein-mediated apoptosis in CD4+ T lymphocytes: A potential mechanism for persistent viral production. J Biol Chem. 2013;288:7626–7644. 10.1074/jbc.M112.408294 23364796 PMC3597804

[43] Llambi F, Moldoveanu T, Tait S, et al A unified model of mammalian BCL-2 protein family interactions at the mitochondria. Mol Cell. 2011;44(4):517–531. 10.1016/j.molcel.2011.10.001 22036586 PMC3221787

[44] Wolf D, Witte V, Laffert B, et al HIV-1 Nef associated PAK and PI3-kinases stimulate Akt-independent Bad-phosphorylation to induce anti-apoptotic signals. Nat Med. 2001;7:1217–1224. 10.1038/nm1101-1217 11689886

[45] Wu X, Dong H, Ye X, et al HIV-1 Tat increases BAG3 via NF-κB signaling to induce autophagy during HIV-associated neurocognitive disorder. Cell Cycle. 2018;13:1614–1623. 10.1080/15384101.2018.1480219 PMC6133340 29962275

[46] Dong H, Ye X, Zhong L, et al Role of FOXO3 activated by HIV-1 tat in HIV-associated neurocognitive disorder neuronal apoptosis. Front Neurosci. 2019;4:44 10.3389/fnins.2019.00044 PMC6369160 30778283

[47] Hassan M, Watari H, AbuAlmaaty A, Ohba Y, Sakuragi N Apoptosis and molecular targeting therapy in cancer. Biomed Res Int. 2014;150845:23 10.1155/2014/150845 PMC4075070 25013758

[48] Fulda S Smac mimetics to therapeutically target IAP proteins in cancer. Int Rev Cell Mol Biol. 2017;330:157–169. 10.1016/bs.ircmb.2016.09.004 28215531

[49] Gao Z, Tian Y, Wang J, et al A dimeric Smac/diablo peptide directly relieves caspase-3 inhibition by XIAP. Dynamic and cooperative regulation of XIAP by Smac/Diablo. J Biol Chem. 2007;282:30718–30727. 10.1074/jbc.M705258200 17724022 PMC3202417

[50] Bertrand MJ, Milutinovic S, Dickson K, et al cIAP1 and cIAP2 facilitate cancer cell survival by functioning as E3 ligases that promote RIP1 ubiquitination. Mol Cell. 2008;30(6):689–700. 10.1016/j.molcel.2008.05.014 18570872

[51] Campbell GR, Bruckman RS, Chu YL, Trout RN, Spector SA SMAC mimetics induce autophagy-dependent apoptosis of HIV-1-infected resting memory CD4+ T cells. Cell Host Microbe. 2018;24(5):689–702. 10.1016/j.chom.2018.09.007 30344003 PMC6250054

[52] Pache L, Dutra MS, Spivak AM, et al BIRC2/cIAP1 is a negative regulator of HIV-1 transcription and can be targeted by smac mimetics to promote reversal of viral latency. Cell Host Microbe. 2015;18(3):345–353. 10.1016/j.chom.2015.08.009 26355217 PMC4617541

[53] Kyei GB, Dinkins C, Davis AS, et al Autophagy pathway intersects with HIV-1 biosynthesis and regulates viral yields in macrophages. J Cell Biol. 2009;186(2):255–268. 10.1083/jcb.200903070 19635843 PMC2717652

[54] Borel S, Robert-Hebmann V, Alfaisal J, et al HIV-1 viral infectivity factor interacts with microtubule-associated protein light chain 3 and inhibits autophagy. AIDS. 2015;29(3):275–286. 10.1097/QAD.0000000000000554 25490467

[55] Boddu P, Carter BZ, Verstovek S, Pemmaraju N SMAC mimetics as potential cancer therapeutics in myeloid malignancies. Br J Haematol. 2019;185(2):219–231. 10.1111/bjh.15829 30836448

[56] Cummins NW, Sainski AM, Dai H, et al Prime, shock, and kill: Priming CD4 T cells from HIV patients with a BCL-2 antagonist before HIV reactivation reduces HIV reservoir size. J Virol. 2016;90:4032–4048. 10.1128/JVI.03179-15 26842479 PMC4810548

[57] Lucas A, Kim Y, Rivera-Pabon O, et al Targeting the PI3K/Akt cell survival pathway to induce cell death of HIV-1 infected macrophages with alkylphospholipid compounds. PLoS One. 2010;5(9):e13121 10.1371/journal.pone.0013121 20927348 PMC2948033

[58] Kim Y, Hollenbaugh JA, Kim DH, Kim B Novel PI3K/Akt inhibitors screened by the cytoprotective function of human immunodeficiency virus type 1 Tat. PLoS One. 2011;6(7):e21781 10.1371/journal.pone.0021781 21765914 PMC3134463

[59] Li P, Kaiser P, Lampiris HW, et al Stimulating the RIG-I pathway to kill cells in the latent HIV reservoir following viral reactivation. Nat Med. 2016;22:807–811. 10.1038/nm.4124 27294875 PMC5004598

